# Type II hereditary angioedema with an apparently de novo SERPING1 mutation in China: A case report and family screening

**DOI:** 10.1097/MD.0000000000047283

**Published:** 2026-01-16

**Authors:** Zepu Guan, Liting Cai, Yang Li, Xinyan She, Buning Ye, Xiaohua Wang

**Affiliations:** a Dermatology Department, Dermatology Hospital of Southern Medical University, Guangzhou, China; b Dermatology Department, Chengdu Second People’s Hospital, Chengdu, China.

**Keywords:** family screening, lanadelumab, SERPING1, type II hereditary angioedema

## Abstract

**Rationale:**

Type II hereditary angioedema (HAE) is a rare and underrecognized condition. Early diagnosis and family screening are essential to prevent life-threatening attacks.

**Patient concerns:**

A 36-year-old woman presented with recurrent facial swelling and dysphagia unresponsive to standard treatments.

**Diagnoses:**

Laboratory analysis revealed decreased C4 and low functional C1 inhibitor (C1-INH) activity with normal antigenic levels, confirming type II HAE. Whole-exome sequencing identified a heterozygous SERPING1 mutation, c.1397G > A (p.Arg466His), classified as pathogenic (ClinVar Accession: VCV000003946.13). Three daughters carried the same variant.

**Interventions:**

The patient received subcutaneous icatibant for acute management and prophylactic lanadelumab. Her daughters were counseled and provided with emergency medication.

**Outcomes:**

Over 7 months of follow-up, the patient remained attack-free without adverse effects. Her daughters were asymptomatic or mildly affected.

**Lessons:**

This case emphasizes the need to consider HAE in unexplained recurrent angioedema and demonstrates the clinical utility of genetic testing and family screening. Lanadelumab was effective and well-tolerated for prophylaxis, consistent with current guideline recommendations.

## 1. Introduction

Hereditary angioedema (HAE) is a rare autosomal dominant disorder characterized by recurrent, self-limiting subcutaneous and submucosal edema that can become life-threatening when involving the larynx.^[1]^ The estimated risk of asphyxiation is 8.6%.^[2]^ Misdiagnosis is common due to overlapping clinical features with allergic angioedema. However, the increased availability of genetic testing and targeted therapies has significantly improved early diagnosis and management outcomes.

## 2. Case report

**Figure 1.**
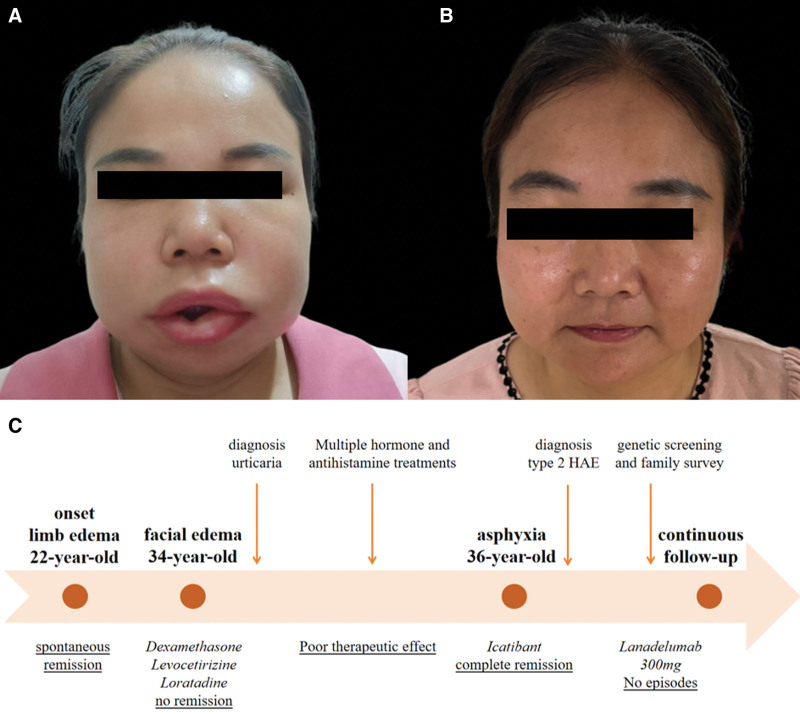
Clinical evolution of proband and the timeline of proband’s episodes. (A) Before treatment, severe asymmetric swelling was observed on the face, eyes, and lips. (B) After treatment, the swelling has completely disappeared. (C) Timeline of proband’s episodes.

A 36-year-old woman presented with a 3-day history of progressive facial swelling and dysphagia. Physical examination revealed marked edema of the periorbital area, lips, and oropharyngeal mucosa (Fig. 1A). Laryngoscopy demonstrated erythematous edema of the epiglottis. She had experienced limb edema during pregnancy 14 years earlier and recurrent swelling of the face and pharynx over the past 2 years, resolving spontaneously within 5 days. Previous treatments with antihistamines, corticosteroids, and epinephrine were ineffective.

Laboratory findings revealed markedly decreased C4 (0.01 g/L; normal 0.16–0.38) and C1-INH functional activity (<7.0%; normal ≥ 58.9) with normal C1-INH antigen levels (199.52 µg/mL; normal 81.46–291.29) and IgE (73.3 IU/mL; normal 0–100). These findings confirmed type II HAE. The patient received subcutaneous icatibant (30 mg), resulting in gradual symptom resolution. Long-term prophylaxis with subcutaneous lanadelumab (300 mg every 2 weeks) was initiated. During 7 months of follow-up, she remained attack-free (Fig. 1B) with no hepatic, renal, or coagulation abnormalities, although her C4 levels remained persistently low (<0.02 g/L). Key disease milestones are presented in Figure 1C.

**Figure 2.**
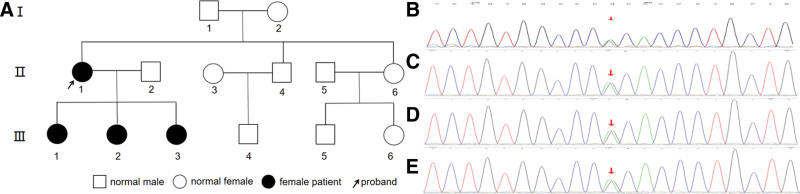
Family tree and sanger sequencing peaks of the proband. (A) Family tree. (B) Sanger sequencing peaks of proband (II 1). (C–E) Sanger sequencing peaks of the proband’s 3 daughters (III 1–3), all with the base missense mutation c.1397G > A (p.Arg466His) in the SERPING1 gene.

Following diagnosis, family screening was performed (Fig. 2A). Dried blood spot (DBS) samples from 3 generations were analyzed using tandem mass spectrometry. Three daughters of the proband exhibited reduced C4 and functional C1-INH activity with normal antigen levels, consistent with type II HAE (Table 1). Whole-exome sequencing identified the same heterozygous SERPING1 variant, NM_000062.3:c.1397G > A (p.Arg466His), in the proband and affected daughters but not in unaffected family members (Fig. 2B–E). The mutation (ClinVar Variation ID: 3946; VCV000003946.13) was classified as pathogenic according to ACMG criteria.^[3]^ As parental samples were not tested, the variant was considered apparently de novo. One daughter reported mild lip swelling triggered by emotional stress, while the others remained asymptomatic. Educational counseling and emergency medication kits were provided.

**Table 1:** Relevant indicators of family members.

Subject	C4 (µg/mL)*		C1-INH Q (µg/mL)†		C1-INH Fun (%)‡		Genetic testing
	Result	Meaning	Result	Meaning	Result	Meaning	Result
I 1	204.21	–	152.57	-	66.25	–	–
I 2	181.24	–	128.51	–	51.64	↓	–
II 1	33.97	↓	199.52	–	<7.00	↓	c.1397G > A (p.Arg466His)
II 2	267.67	–	221.89	–	80.59	–	–
II 4	242.43	–	164.94	–	68.01	–	–
II 5	206.47	-	169.57	–	74.58	–	–
III 1	30.95	↓	200.79	–	<7.00	↓	c.1397G > A (p.Arg466His)
III 2	12.92	↓	209.75	–	<7.00	↓	c.1397G > A (p.Arg466His)
III 3	37.13	↓	232.16	–	<7.00	↓	c.1397G > A (p.Arg466His)

* Normal range: 72.85 to 372.95 µg/mL.

† Normal range: 81.46 to 291.29 µg/mL.

‡ Normal range: ≥58.9%.

–: Normal, ↑: Rising, ↓: Declining.

C1-INH Fun = C1-INH functional, C1-INH Q = C1-INH quantitative.

## 3. Methods

Sample collection and biochemical assays: DBS samples were collected from the proband and family members during an attack-free period. DBS samples were air-dried for at least 4 hours at room temperature and stored at 4℃ before analysis. C4 concentration, C1-INH antigenic levels, and C1-INH functional activity were simultaneously measured using tandem mass spectrometry (MS/MS) at Suzhou Ruifudi Medical Laboratory, China. According to the laboratory reference criteria, functional C1-INH activity < 50% of the normal value is diagnostic for HAE type I/II. Reference ranges provided by laboratory were as follows: C4 (72.85–372.95 µg/mL), C1-INH antigen (81.46–291.29 µg/mL), and C1-INH function (≥58.9%).

Genetic testing: whole-exome sequencing was performed by BGI Genomics using the GenCap® Whole-Exome Capture Kit V6.0 and sequenced on the Illumina NovaSeq 6000 platform (Illumina, San Diego). Variant annotation was performed using ClinVar, HGMD, and gnomAD databases. The variant was confirmed by Sanger sequencing.

Family screening: Family cascade screening was conducted using DBS samples analyzed by tandem mass spectrometry for C4, C1-INH antigen, and functional activity. Individuals with abnormal biochemical results were further evaluated clinically and genetically.

## 4. Discussion

HAE is characterized by recurrent, self-limiting cutaneous and submucosal swelling, which may involve the gastrointestinal tract or the airway.^[1]^ It is unresponsive to antihistamines or corticosteroids. The global prevalence of HAE is estimated to range from 1:50,000 to 1:100,000. Based on C1-INH status, HAE is classified into 3 types: type I (reduced antigen, ~85%), type II (reduced function, ~15%), and type III (normal C1-INH). Although laryngeal attacks account for only approximately 0.9% of episodes, over half of HAE patients experience laryngeal involvement at least once in their lifetime, and this may be the presenting symptom.^[4–6]^ In Chinese patients, the incidence of laryngeal attacks has been reported as about 58.9%, with mortality rates as high as 11% to 40%.^[7]^ These findings underscore the importance of early recognition and timely intervention.

In China, only 1.27% of reported HAE cases are classified as type II, which is far lower than global estimates and likely reflects underdiagnosis.^[7]^ Diagnostic delays are common: the average latency to diagnosis in China is 12.64 years, similar to that reported in Spain and Denmark. Such delays are particularly problematic given the potential severity of untreated HAE.^[7–9]^ For patients with suggestive symptoms, measurement of C4 and of C1-INH antigen and functional activity is essential. In our patient, persistently low C4 despite 7 months of lanadelumab treatment supports the utility of C4 as a screening marker. Differential diagnosis should also include acquired C1-INH deficiency, particularly in older patients (>40 years), in those with a negative family history, or when hematologic or autoimmune disease is present.^[10]^

Traditional therapies such as tranexamic acid, danazol, and fresh-frozen plasma have limited efficacy and carry significant adverse effects. Current management comprises both on-demand and prophylactic approaches. On-demand options – C1-INH concentrate, ecallantide, and icatibant – provide rapid symptom relief.^[11]^ For long-term prophylaxis, plasma-derived C1-INH, lanadelumab, and berotralstat are recommended.^[11]^ In the present case, icatibant was used for the acute episode, followed by lanadelumab, which achieved complete disease control without observed side effects. While encouraging, this single-patient experience should be interpreted cautiously and regarded as supportive of existing evidence rather than definitive proof of population-level efficacy.

Reports of type II HAE in Chinese populations remain scarce, and documentation of family screening is limited. WAO/EAACI guidelines recommend screening all first-degree relatives irrespective of symptoms.^[5]^ One study found that undiagnosed HAE patients had a 2.7-fold higher mortality risk and markedly reduced life expectancy (40.8 years vs 72.0 years) compared with diagnosed individuals. Early identification and education of asymptomatic carriers are therefore crucial. Although HAE follows an autosomal dominant inheritance pattern, approximately 25% of cases arise from de novo mutations.^[12]^ Among the 748 known type II HAE mutations, 70% involve substitution of arginine 466 with cysteine or histidine, rendering C1-INH nonfunctional.^[12]^ In our case, we identified a c.1397G > A (p.Arg466His) mutation in the proband; subsequent family screening detected 3 additional affected individuals, enabling early intervention and potentially reducing the risk of fatal complications.

## 5. Conclusion

This case illustrates the diagnostic complexity of type II hereditary angioedema and the value of genetic confirmation. The heterozygous SERPING1 mutation NM_000062.3:c.1397G > A (p.Arg466His), classified as pathogenic, was apparently de novo. Early identification through genetic testing and family screening enabled timely counseling and prophylactic management. Lanadelumab provided effective disease control, consistent with international guidelines.

## Acknowledgments

We thank our patients for granting permission to publish this information.

## Author contributions

**Conceptualization:** Zepu Guan, Xiaohua Wang.

**Data curation:** Liting Cai, Yang Li.

**Formal analysis:** Xinyan She.

**Investigation:** Buning Ye.

**Writing – original draft:** Zepu Guan, Xiaohua Wang.

**Writing – review & editing:** Zepu Guan, Xiaohua Wang.
